# Colostrum avoidance and associated factors among mothers having children less than 2 years of age in Aksum town, Tigray, Ethiopia: a cross-sectional study 2017

**DOI:** 10.1186/s13104-018-3712-z

**Published:** 2018-08-20

**Authors:** Girmay Teklay Weldesamuel, Hagos Tasew Atalay, Teklewoini Mariye Zemichael, Hadgu Gernsea Gebre, Dawit Gebrezgiabher Abraha, Awoke Kebede Amare, Eskedar Birhanie Gidey, Tsega Teshale Alemayoh

**Affiliations:** 1 School of Nursing, Aksum University, Po. Box 298, Aksum, Tigray Ethiopia; 2 Department of Medical Laboratory, Aksum University, Aksum, Tigray Ethiopia

**Keywords:** Pre-lacteal, Antenatal care, Postnatal, Information, Ethiopia

## Abstract

**Objective:**

To assess colostrum avoidance practices and associated factors among mothers of children aged less than two in Aksum town, Tigray, Ethiopia, 2017.

**Result:**

Colostrum avoidance was practiced by 6.3% (95% CI = 4.2%, 8.6%) of mothers having children aged < 24 months in Aksum town. In a multivariate logistic regression analysis at *P* value of < 0.05, lower number of antenatal care visit (AOR = 4.45, 95% CI = 1.9, 10.5), lack postnatal follow up [AOR = 2.98 (95% CI = 1.22, 7)] and poor maternal level of information on colostrum feeding [AOR = 4.8 (95% CI = 1.83, 12.69)] were statistically associated with colostrum avoidance. Coordination, strengthening and sustaining of awareness creation strategies and approaches is recommended for the promotion of the nutritional value of colostrum and its health benefits.

## Introduction

Breastfeeding is the fundamental rights of the child and the most important for child survival, prevention of childhood infections and optimal nutrition for early life [1]. Colostrum is the primary milk created in the first few days after birth. It is a normative standard for the infants regarding the complete form of nutrition [2] which is considered as the “golden milk” [3]. Furthermore, colostrum has a positive effect in the prevention of childhood malnutrition [4] which also delivers natural immunity (baby’s first immunization) against many bacteria and viruses through establishing important bacteria in the baby’s gut [5].

In the developing and least developing countries, breastfeeding mothers have lack of awareness about colostrum feeding [6, 7], thus prevent their infants from colostrum’s feeding. Avoiding colostrum in the first three crucial days after birth increases the risk of infection and death among neonates [8]. Child mortality in developing countries is more associated with malnutrition [9]. Colostrum avoiding deprives the newborns of nutrients and immunoglobulins and causing a reduction in the priming of the gastrointestinal tract, and increases the risk of infant morbidity and mortality [10]. The risk of infant death as a result of infection increases with increasing of pre-lacteal feeding and delaying of breastfeeding [11]. Colostrum deprivation was also the major cause of stunting in children [12].

Although colostrum feeding provides newborns with immunity to infection, any practice that reduces a frequency or volume of breastfeeding during this time could reduce an infant’s long-term health and immunological defense [11].

Mothers who reside in developing countries avoid their colostrum due to traditional beliefs [13, 14], prolonged labor, surgical deliveries and neonatal illness are also the hindrance of colostrum’s feeding [15], cesarean delivery [16], and lack of knowledge regarding the benefits of colostrum’s feeding [17].

The magnitude of colostrum avoidance has varied in different developing countries like 13.5% in Ethiopia [18], 76% in Jammu and Kashmir [19] and 85.7% in Jaipur city [13]. Therefore, preventing the practice of colostrum avoidance in the first three crucial days can help to prevent neonatal morbidity and mortality [20].

Even though the world health organization (WHO) recommended to initiate colostrum feeding within the first hour of birth, a higher number of mothers avoided their colostrum before giving milk to their infant [21]. Although Ethiopia has developed the National Infant and Young Child Feeding (IYCF) guideline which recommends colostrum feeding [22], colostrum avoidance is still practiced in many parts of Ethiopia including Tigray region.

The problem of colostrum avoidance has been an alarming concern for the ministry of health for years. Even though colostrum avoidance is widely practiced, there is a scarcity of information on factors related to colostrum avoidance in the Tigray regional state, particularly in Aksum town. The result of this study will give important update information for health service providers, policymakers and program managers to design intervention strategies in the promotion of optimal breastfeeding practices.

## Main text

### Study design and setting

A community-based, cross-sectional study design was employed in this study. The data collection was taken for a month in March 2017. Aksum town locates in Tigray region of Ethiopia and 1024 km far from the Addis Ababa which is capital city of the country. Aksum town has a total population of 56,576. It has one general hospital, one referral hospital, two health centers and seven private clinics.

### Sample size determination

The sample size was calculated using single population proportion formula by considering both magnitude and associated factors with the highest sample under the following parameters: 13.5% prevalence of colostrum discarding in Raya Kobo district [18] and a 4% margin of error with 95% confidence level. The required final sample size with the design effect of 1.5 and 10% non-response rate was 484.

### Sampling technique

Multi-stage sampling technique was employed to select 484 study participants. To obtain the sample size from every three kebeles proportional allocation to sample size was done. Systematic sampling technique (k = 8) was used to choose the household for an interview and the starting mother was selected using a lottery method.

### Data collection tools and procedure

Data was collected using semi-structured questionnaires by six diploma midwives and three Bachelor of Science degree holder. The tool was adapted, modified contextually from the Ethiopian Demographic and Health Survey [8], the Ethiopian National Nutrition Program [23] and from the research done in the Raya Kobo district [18].

### Operational definition

*Good level of information about breastfeeding* Those mothers who answer two or more components of breastfeeding counseling during their ANC visit [24].

*Poor level of information about breastfeeding* Those mothers who answer one or none components of breastfeeding counseling during their ANC visit [24].

*Components of breastfeeding counseling* during their ANC visit: Benefits of breastfeeding, the positioning of the baby, exclusive breastfeeding, management of breast problem and expression of breast milk.

### Data quality assurance

Training and orientation were given for data collectors. The questionnaire was initially prepared in English and then translated into Tigrigna version (local language) by different experts of both languages to check its consistency. The questionnaire was pre-tested on 5% of the sample size in shire town. The collected data were reviewed and checked for completeness and consistency by the supervisor and principal investigator on a daily basis.

### Data processing and analysis

The data were coded, entered, cleaned, and edited using EPIDATA version 3.1 and then exported into SPSS Version 22.0 for analysis. Binary logistic regression analysis was employed to examine the statistical association between the outcome variable and every single independent variable. Variables which showed statistical significance during bivariate analysis at ≤ 20% (p-value ≤ 0.20) were entered into multivariate logistic regression to isolate an independent effect of the predictors by using the backward elimination method. The Hosmer–Lemenshow test was used to check the appropriateness of the model for analysis. Results were presented using tables and texts. Adjusted odds ratios (AOR) with 95% CI, were estimated to assess the strength of associations and statistical significance was declared at a p-value < 0.05.

## Results

### Socio-demographic characteristics

In this study, 477 mothers having children less than 2 years of age were interviewed with a response rate of 98.5%. From the total respondents, 202 (42.3%) were aged from 25 to 29 years old and 319 (66.9%) had ≥ 4 family size. Majority of the respondents 407 (85.3%) were married, 196 (41.1%) were secondary school and above and 396 (83%) of the respondents were orthodox Christian. Out of the total respondents, 291 (61%) were housewives by occupation and 393 (82.4%) of the mothers had ≤ 3 children in number. Regarding the children’s condition, 254 (53.2%) of the children were male, 145 (30.4%) were aged less than 6 months and 239 (50.1%) of the children were in the birth order of 2–3 with 212 (44.4%) birth spacing of greater than 24 months (Table 1).

**Table 1:** Socio-demographic characteristics among mothers having children less than 2 years of age in Aksum town, Tigray, Ethiopia, 2017

Demographic variables	Frequency (n = 477)	Percentage (%)
Age of the mother		
≤ 19	18	3.8
20–24	97	20.3
25–29	202	42.3
30–34	85	17.8
35–39	53	11.1
40–44	17	3.6
≥ 45	5	1
Family size		
≤ 3	158	33.1
≥ 4	319	66.9
Level of educational		
No education	161	33.8
Primary school (1–8)	120	25.2
Secondary school and above	196	41.1
Marital status		
Single	17	3.6
Married	407	85.3
Widowed	43	9
Divorced	10	2.1
Religion		
Orthodox	396	83
Muslim	81	17
Ethnicity		
Tigray	473	99.2
Amhara	4	0.8
Occupation		
Housewife	291	61
Governmental employee	59	12.4
Private employee	91	19.1
Daily labor	35	7.3
Other	1	0.2
Number of children		
≤ 3	393	82.4
≥ 4	84	17.6
Age of the child		
< 6 months	145	30.4
6–11 months	141	29.6
12–17	89	18.7
18–24	102	21.4
Sex of the child		
Male	254	53.2
Female	223	46.8
Birth order		
Birth order one	150	31.4
Birth order 2–3	239	50.1
Birth order ≥ 4	88	18.4
Birth spacing		
No previous child	150	31.4
< 24 months	115	24.1
≥ 24 months	212	44.4

#### Colostrum avoidance practice and health care service utilization

In the post-delivery result, 6.3% (95% CI 4.2%, 8.6%) of mothers were avoiding their colostrum in the first 5 days and 13 (43.3%) of them were due to maternal medical illness. Regarding the breastfeeding initiation, 271 (56.8%) mothers have initiated breastfeeding within 1 h and 48 (10.1%) were providing pre-lacteal feeding within 3 days before giving breastfeeding to their child. In this study, 461 (96.6%) mothers were attended ANC visit, and 341 (71.5%) of them were utilized four times and above (which is internationally recommended) and 467 (97.9%) of them have been gotten breastfeeding counseling at ANC clinic. About 412 (86.4%) mothers had at least one visit to the PNC and all of them have been gotten breastfeeding counseling in the post-natal clinic. Regarding the maternal level of information on colostrum feeding about 447 (93.7%) mothers had information on the advantage of colostrum giving to their child. About 434 (91%) mothers were at a good level of information by which they could mention two or more components of breastfeeding counseling during their ANC visit (Table 2).

**Table 2:** Colostrum avoidance practice and health care service utilization among mothers having children less than 2 years of age, in Aksum Town, Central Zone of Tigray, 2017

Variables	Frequency	Percentage (%)
Colostrum avoided (477)		
Yes	30	6.3
No	447	93.7
Reason for avoiding colostrum (30)		
Maternal medical illness	13	43.3
For the child growth	4	13.3
My breast has no milk	11	36.7
Cause abdominal discomfort and diarrhea	2	6.7
Did you give pre-lacteal feeding?		
Yes	48	10.1
No	429	89.9
Reason to give pre-lacteal (48)		
Breast feed for infant will cause thirsty	13	27.1
For child growth	3	6.3
Breast problem	16	33.3
Inadequate breast milk secretion	2	4.2
Infant feeding problem	3	6.3
Maternal medical illness	10	20.8
Cultural practice	1	2.1
Breast feeding initiation (477)		
Within 1 h	271	56.8
Greater than 1 h	206	43.2
ANC visit (477)		
Yes	461	96.6
No	16	3.4
How many (477)		
≥ 4	341	71.5
< 4	136	28.5
Breast feeding counseling (477)		
Yes	467	97.9
No	10	2.1
About what did you counseled (multiple answer was possible)? (467)		
Benefit of breast feeding	408	85.5
Position of the baby	296	62.1
Exclusive breast feeding	388	81.3
Management of breast feed problem	243	50.9
Expression of breast milk	306	64.2
Place of giving birth (477)		
Health facility	453	95
At home	24	5
Mode of delivery (477)		
C/s delivery	41	8.6
Normal	436	91.4
The person assisted you during delivery (477)		
Health profession	453	95
Traditional birth attendant	24	5
PNC follow (477)		
Yes	412	86.4
No	65	13.6
Maternal level pf information		
Good	434	91
Poor	43	9

#### Factors associated with colostrum avoidance practice

In the binary logistic regression at a p-value of ≤0.2, child age, a number of ANC visit, breastfeeding counseling during ANC visit, place of delivery, PNC follow up, maternal level of information was statistically associated with colostrum discarding. In multiple logistic regression by using backward elimination method, mothers who have lower number of antenatal care visit were 4.45 higher to avoid their colostrum than those who had four or more ANC follow up (AOR = 4.45, 95% CI = 1.9, 10.5), mothers with lack of postnatal follow up were 2.98 times more to practice colostrum avoidance than their counterpart [AOR = 2.98 (95% CI = 1.22, 7)] and the odds of mothers with poor maternal level of information on colostrum feeding were 4.8 times more practiced colostrum avoidance [AOR = 4.8 (95% CI = 1.83, 12.69)] were statistically associated with colostrum avoidance (Table 3).

**Table 3:** Factors associated with colostrum avoiding among mothers having children less than 2 years of age, in Aksum town, central zone of Tigray, 2017

Variables	Colostrum avoiding		Crude OR (CI 95%)	Adjusted OR (CI 95%)
	Yes	No		
Number of ANC visit				
≥ 4	10 (2.9%)	331 (97.1%)	1	1
< 4	20 (14.7%)	116 (85.3%)	5.70 (2.595, 12.549)	4.45 (1.9, 10.5)*
Breastfeeding counseling during ANC				
Yes	26 (5.6%)	441 (94.4%)	1	1
No	4 (40%)	6 (60%)	11.30 (3, 42.56)	0.79 (0.1, 5.87)
Place of delivery				
Heath facility	23 (5.1%)	430 (94.9%)	7.70 (2.9, 20.41)	1
At home	7 (29.2%)	17 (70.8%)		2.99 (0.97, 9.3)
PNC follow up				
Yes	19 (4.6%)	393 (95.4%)	14.213 (1.9, 9.33)	1
No	11 (16.9%)	54 (83.1%)		2.9 (1.2, 7.0)*
Maternal level of information:				
Good	21 (4.8%)	413 (95.2%)	1	1
Poor	9 (20.9%)	34 (79.1%)	5.2 (2.213, 12.248)	4.8 (1.83,12.69)*

*Statistical association (p < 0.05)

## Discussion

This study was aimed to assess colostrum avoidance and associated factors among mothers having children less than 2 years of age in Aksum town. This study revealed that the prevalence of colostrum avoidance was 6.3% (95% CI 4.2%, 8.6%) which is comparable to the study reported from Kersa district [25]. This study is lower than studies done in different corners of Ethiopia (studies were done in the Raya Kobo district [18], Afambo District [26], Amibara district [27], Goba Woreda [28] and rural Northern Ethiopia [11].) The current finding is also lower than the reports from other developing countries (76% in India [19], 82% in Kuwaiti [29]. Such a difference could be due to the difference in infant feeding styles and socio-cultural practices across the communities and the time of the studies done. This could also be the place of residence and difference in maternal educational level. Hence, mothers who reside in the towns would have better access to maternal and child health services.

Antenatal care helps mothers to increase the awareness of the advantage of colostrum feeding. The odds mothers having less than four ANC visit were 4.45 times more avoided their colostrum than their counterpart. In most women, ANC visit may offer a good educational channel. Participation in a discussion at ANC may increase awareness mothers on the importance of colostrum to the neonate.

Even though the proportion of immunological substances in colostrum is greatest in the first 3 days after birth [30], the odds of colostrum avoiding in mothers with lack of postnatal follow up were 2.9 times higher than mothers who had a postnatal follow-up. This could lead to the reduction of the priming of the gastrointestinal tract and increases the risk of infant morbidity and mortality [10]. A cesarean section may also hamper the immediate colostrum feeding due to the post-operative efforts [31].

In our study, as the level of information of the mothers increase the odds of colostrum avoiding decrease. The odds of mothers with poor level information on colostrum feeding were 4.8 times higher to avoid their colostrum as compared with mothers who have a good level of information about colostrum feeding. This is consistent with the study done in the Raya Kobo district in which mothers who had less awareness of the benefits of colostrum were more likely to avoid colostrum compared with mothers who were aware of the advantages of colostrum for infants [18]. Awareness creation on the advantage of colostrum’s feeding through health extension worker would be important for increasing the maternal level of information.

## Conclusion

Maternal factors were the contributing factors for practicing of colostrum avoiding in Aksum town. Coordination, strengthening and sustaining of the awareness creation strategies and approaches were recommended for the promotion of the nutritional value of colostrum and its health benefits.

## Limitation

This study shares some limitations. The limitation of this study was the information obtained from mothers might be subjected to recall bias. Lack of support with qualitative data is also another limitation. The study also shares the limitation of the cross-sectional study design.

## Abbreviations

AOR: adjusted odd ratio
ANC: antenatal care
COR: crudes odd ratio
IYCF: infant and young child feeding
SPSS: Statistics Package for Social Science
PNC: postnatal care
WHO: World Health Organization
